# Continuous Glucose Monitor Failure Following Exposure to High‐Output LED‐Based Photography

**DOI:** 10.1111/ddg.70175

**Published:** 2026-02-15

**Authors:** Lennart Ocker‐Serger, Nessr Abu Rached, Karim Ahmed, Carolin Frost, Falk G. Bechara

**Affiliations:** ^1^ Department of Dermatology Allergology and Venerology Ruhr‐University Bochum, Germany; ^2^ Department of Dermatology University Hospital Essen, Germany

Dear Editors,

Transcutaneous continuous glucose monitors (CGMs) are widely used by patients with diabetes for real‐time glycemic control and have demonstrated resilience under common clinical conditions, including MRI and CT imaging.^1^ However, no data exist regarding their performance during high‐intensity light‐based imaging procedures. Automated total‐body photography uses synchronized high‐output LED flashes to capture 3D skin surface maps for dermatologic monitoring.^2^ **^,^** ^3^ We report a series of patients who experienced abrupt CGM sensor malfunction immediately following whole‐body LED‐based imaging. To our knowledge, this represents the first published report of such an event and highlights a potential interaction between dermatologic imaging technologies and digital glucose monitoring systems.

We reviewed six consecutive cases between March and May 2025 in which patients with type 1 or type 2 diabetes using CGM devices experienced acute sensor failure following a single session of whole‐body photography using the Vectra^®^ WB360 imaging system (Canfield Scientific Inc., Parsippany). Clinical details, including sensor models, placement, and malfunction characteristics, are summarized in Table 1. Relevant patient information was obtained through structured chart review and direct interviews, including demographics, diabetes type, sensor age, and timing of malfunction. The manufacturer of the imaging system was contacted, and per manufacturer report, the proper functioning of the unit, including its intended LED‐based illumination mechanism and operational integrity, was confirmed. During the same three‐month period, 345 whole‐body photography sessions were performed in our department, six of which involved patients wearing CGM systems. Given the very small number of CGM users in this cohort, we refrained from calculating a malfunction rate, as it would not provide a meaningful or interpretable metric.

**TABLE 1:** Characteristics of patients with CGM malfunction following Vectra WB360 whole‐body photography. Patients 2 and 4 were wearing two different CGM systems simultaneously, one on each upper arm, at the time of imaging. In patient 2, only one sensor exhibited malfunction, while the second sensor remained unaffected. In contrast, both sensors in patient 4 failed simultaneously.

Case	Age	Sex	Diabetes Type	CGM Model	Sensor Age (days)	Location	Time to Malfunction	Type of Malfunction	Resolution
1	64	M	2	FreeStyle Libre 3 Plus	4	Upper arm	Within minutes	No data transmission, system error	Replaced by manufacturer
2a	53	F	1	Medtronic simplera Sync	2	Upper arm (both)	Within minutes	No data transmission, system error	Replaced by manufacturer
2b				Dexcom G6	3		No malfunction reported	Not applicable	Not applicable
3	80	M	1	FreeStyle Libre 3	9	Upper arm	Within minutes	No data transmission, system error	Replaced by patient
4a	25	F	1	FreeStyle Libre 3 Plus	7	Upper arm (both)	Within minutes (both)	No data transmission, system error (both)	Replaced by patient (both)
4b				Medtronic simplera Sync	2				
5	56	M	1	FreeStyle Libre 3	13	Upper arm	Within minutes	No data transmission, system error	Replaced by patient
6	62	M	NA (lifestyle)	FreeStyle Libre 3	4	Upper arm	Within minutes	No data transmission, system error	Replaced by patient

All patients reported device malfunction, manifesting as abrupt data loss or system errors, within minutes after imaging. Notably, two patients were wearing two CGM systems simultaneously. One of these experienced a dual sensor failure (patient 4), while the other experienced failure in only one device (patient 2). All sensors were within their active wear period and exhibited no mechanical damage. Sensor replacement was required in all malfunctioning cases.

CGM systems function via an enzymatic electrochemical mechanism, translating interstitial glucose levels into electrical signals.^4^ These devices have shown resilience in high‐radiation environments such as MRI and CT without loss of function,^1^ but they are not routinely tested under intense light bursts. Several hypotheses may explain the observed malfunctions. First, the possibility of transient electromagnetic interference generated during LED flash discharge may disrupt signal transmission, as some CGM systems list electromagnetic susceptibility in regulatory documentation. Manufacturer instructions for use for multiple CGM systems caution against exposure to strong electromagnetic fields or radiofrequency emissions, noting possible performance degradation or device damage.^5^ While the Vectra WB360 employs LED flashes rather than MRI or CT technology, its high‐power discharge may generate brief electromagnetic emissions within the sensitivity range described by CGM manufacturers. Second, although CGMs do not rely on optical sensors, high‐intensity light exposure may interact with exposed components or housings to create electrical noise or interference. Lastly, although thermal effects seem unlikely given brief pulse durations, cumulative local heating or photothermal effects on electrode materials cannot be fully excluded. Importantly, all CGM sensors were covered by their standard adhesive patches during imaging, making a direct light‐induced effect unlikely and further supporting electromagnetic interference as a more plausible explanation.

These findings suggest that even under proper use conditions, specific environmental factors during high‐intensity total‐body imaging may interfere with CGM functionality. With total‐body photography becoming increasingly central in early skin cancer detection and CGM use rising rapidly in diabetes care, further technical evaluation and manufacturer‐led investigations are essential to ensure safe co‐usage.

## CONFLICTS OF INTEREST STATEMENT

None.
